# Insulin Resistance in Relation to Lipids and Inflammation in Type-2 Diabetic Patients and Non-Diabetic People

**DOI:** 10.1371/journal.pone.0153171

**Published:** 2016-04-13

**Authors:** Ying-Mei Feng, Dong Zhao, Ning Zhang, Cai-Guo Yu, Qiang Zhang, Lutgarde Thijs, Jan A. Staessen

**Affiliations:** 1 Department of Endocrinology, Lu He Hospital and Beijing Key Laboratory of Diabetes Prevention and Research, Capital Medical University, Beijing, China; 2 Studies Coordinating Centre, Research Unit Hypertension and Cardiovascular Epidemiology, KU Leuven Department of Cardiovascular Diseases, University of Leuven, Leuven, Belgium; 3 R&D Group VitaK, Maastricht University, Maastricht, the Netherlands; Innsbruck Medical University, AUSTRIA

## Abstract

**Background:**

We demonstrated in experimental studies that hypercholesterolaemia enhances the proliferation of haematopoietic stem cells and the subsequent differentiation to neutrophils, whereas HDL-cholesterol inhibits these processes. To translate our experimental findings to clinical practice, we investigated in Chinese type-2 diabetic patients and in Flemish non-diabetic people the independent and joint associations of insulin resistance with markers of dyslipidaemia and inflammation, while looking for consistency between ethnicities and across the spectrum of insulin resistance.

**Methods:**

We studied 798 Chinese patients with type-2 diabetes (53.6% women; mean age, 60.6 years) admitted to a tertiary referral centre and 1060 white Flemish (50.5%; 51.1 years) randomly recruited in Northern Belgium. Fasting insulin resistance (HOMA-IR) was derived from C-peptide in Chinese and from insulin in Flemish using the Homeostasis Model of Assessment algorithm. In multivariable-adjusted analyses, HOMA-IR was regressed on triglycerides, HDL-cholesterol and neutrophil count.

**Results:**

In Chinese patients, the percentage changes in HOMA-IR associated with triglycerides, HDL-cholesterol and neutrophils (per 1-SD increment) amounted to 8.1 (95% confidence interval, 3.0 to 13.4; p = 0.0015), -8.7 (-13.0 to -4.2; p = 0.0002) and 5.6 (1.0 to 10.4; p = 0.017). In non-diabetic Flemish, the corresponding estimates were 11.7 (8.3 to 15.1; p<0.0001), -1.7 (-4.6 to 1.4; p = 0.28) and 3.3% (0.5 to 6.3; p = 0.022), respectively. None of the interaction terms between the three explanatory variables reached significance in Chinese or Flemish (p≥0.10).

**Conclusions:**

Insulin resistance increases with the serum level of triglycerides and the blood neutrophil count, but decreases with serum HDL-cholesterol concentration. These associations were consistent in Chinese type-2 diabetic patients and non-diabetic Flemish people and were independent from one another. The clinical implications are that future studies should focus on intervening with serum triglyceride and HDL-cholesterol levels or controlling inflammation as a way to prevent or treat insulin resistance.

## Introduction

Insulin resistance refers to the deficient regulation by insulin of energy substrate utilisation in peripheral tissues. It is a major risk factor in the pathogenesis of type-2 diabetes mellitus.[1] Insulin resistance is characterised by dyslipidaemia, as exemplified by high triglyceride levels and low concentration of high-density lipoprotein (HDL) cholesterol, the most likely underlying cause being the increased free fatty acid flux secondary to insulin resistance.[1] Furthermore, experimental research[2, 3] and human studies[4] established that inflammatory processes either contribute to insulin resistance, in particular in the presence of obesity, or evolve as a consequence of the metabolic dysregulation associated with insulin resistance.[5] In keeping with the literature,[2, 3, 5] we recently demonstrated in experimental studies[6] that hypercholesterolaemia enhances the proliferation of haematopoietic stem cells and their subsequent differentiation to neutrophils, whereas HDL-cholesterol inhibits these processes. These findings support the role of low-grade inflammation as a pathogenetic mechanism in diabetes mellitus and its complications.[7]

Previous clinical studies of the association of insulin resistance with markers of inflammation[7–10] or dyslipidaemia included middle-aged overweight or obese participants,[11–13] people at risk of diabetes mellitus,[9–11] or patients with the metabolic syndrome[11–13] or type-2 diabetes.[14, 15] These studies were small[15] or confined to selected volunteers[13] or to a specific ethnic group.[8] To dissect the independent associations of insulin resistance with markers of dyslipidaemia and inflammation and to translate our experimental findings,[6] we studied insulin resistance in relation to serum triglycerides and HDL-cholesterol and the blood neutrophil count in Chinese patients with type-2 diabetes mellitus and in non-diabetic people randomly recruited from a Flemish population.

## Methods

### Study populations

The Chinese and Flemish studies complied with the Helsinki Declaration for investigation of human subjects. They received ethical approval from the competent Institutional Review Boards of the Capital Medical University and the Faculty of Medicine of the University of Leuven. All participants provided written informed consent.

From 2006 until 2011, 1054 diabetic patients were admitted to the Department of Endocrinology at Lu He hospital in Beijing. According to the diagnostic criteria of the American Diabetes Association, 946 had type-2 diabetes and were eligible for analysis. They all had a plasma glucose of at least 7.0 mmol/L while fasting or of 11.0 mmol/L or more 2 hours after an orally administered glucose load of 75 gram. We excluded 148 patients from analysis, because not all required measurements were available on the database (n = 117) or because of extreme values of variables exceeding the mean by 3 SDs or more (n = 31). Thus the number of Chinese diabetic patients statistically analysed totalled 798. Recruitment for the Flemish Study on Environment, Genes and Health Outcomes (FLEMENGHO) started in 1985 and continued until 2004.[16] The initial participation rate was 78.0%. The participants were repeatedly followed up. From May 2005 until June 2014, we mailed an invitation letter to 2115 former participants for a follow-up examination. However, 303 were unavailable, because they had died (n = 70), because they had been institutionalised or were too ill (n = 60), or because they had moved out of the area (n = 173). Of the remaining 1812 former participants, 1352 renewed informed consent. The participation rate was therefore 74.6%. We excluded 292 participants from analysis, because they had diabetes (n = 49) defined as a fasting plasma glucose of 7.0 mmol/L or higher, because fasting insulin or glucose were not available in the database (n = 231), or because measurements deviated more than 3 SDs from the mean (n = 12). The analyses therefore included 1060 non-diabetic FLEMENGHO participants (S1 Fig).

### Clinical measurements

In both studies, trained observers measured blood pressure to the nearest 2 mm Hg by auscultation of the Korotkoff sounds, using a standard mercury sphygmomanometer. Blood pressure was the average of three readings in Lu He patients and five in FLEMENGHO participants. Mean arterial pressure was diastolic pressure plus one third of pulse pressure. Hypertension was a blood pressure of at least 140 mmHg systolic or 90 mmHg diastolic or use of antihypertensive drugs. The observers measured each participant’s anthropometric characteristics and collected information on medical history, smoking and drinking habits, and intake of medications, using structured interviews in Lu He patients or standardised questionnaires in FLEMENGHO. Body mass index was weight in kilograms divided by the square of height in meters.

### Biochemical measurements

With participants fasting, venous blood samples were drawn for measurement of the total and differential white blood cell count, serum total cholesterol, high-density lipoprotein (HDL) cholesterol, triglycerides, and creatinine, plasma glucose, and serum levels of C-peptide or insulin. Insulin resistance was computed by Homeostasis Model Assessment (HOMA-IR; http://www.dtu.ox.ac.uk/HOMAcalculator/), using C-peptide in diabetic patients and insulin in non-diabetic people. Glomerular filtration rate (eGFR) was derived from serum creatinine by the Chronic Kidney Disease Epidemiology Collaboration (CKD-EPI) equation.[17] Low-density lipoprotein (LDL) cholesterol was computed from serum total and HDL-cholesterol and serum triglycerides by the Friedewald equation.[18] Participants were classified as having dyslipidaemia if at least one of the following criteria was met: total cholesterol higher than 4.9 mmol/L, LDL-cholesterol exceeding 3 mmol/L, triglycerides higher than 1.7 mmol/L or HDL-cholesterol less than 1.2 mmol/L in women and 1 mmol/L in men.[19]

### Statistical analysis

For database management and statistical analysis, we used the SAS system, version 9.3 (SAS Institute Inc., Cary, NC). Significance was a two-tailed α-level of 0.05 or less. We normalised the distributions of HOMA-IR, C-peptide and insulin by a logarithmic transformation. For untransformed and logarithmically transformed variables, we expressed the central tendency and spread of the distributions by the arithmetic mean and SD and the geometric mean and interquartile range, respectively. We compared means by the large-sample z-test and proportions by Fisher’s exact test. In unadjusted analyses, we explored trends of variables across thirds of the distributions of HOMA-IR, C-peptide and insulin. We searched for covariables of the indexes of insulin resistance using a stepwise regression procedure with the p-values for variables to enter and stay in the models set at 0.15. The covariables considered were sex, age, body mass index, mean arterial pressure, use of antidiabetic drugs (insulin, sulphonylurea, metformin and α-glucosidase inhibitors), lipid-lowering drugs (statins, niacin and fibrates), antihypertensive drugs (diuretics, β-blockers, calcium-channel blockers and inhibitors of the renin-angiotensin system [angiotensin-converting enzyme inhibitors and angiotensin receptor blockers]) and nonsteroidal anti-inflammatory drugs including aspirin. We standardised the indexes of insulin resistance to the average in the population (mean or ratio) of significant covariables identified by stepwise regression. While accounting for covariables by standardisation, we regressed the indexes on insulin resistance on serum triglycerides and HDL-cholesterol and the neutrophil count, first considering each of these three variables separately and next introducing all three together in the same model. Finally, we searched for interaction between these three variables.

## Results

### Characteristics of participants

In all 798 Lu He patients (53.4% women), age averaged (SD) 60.6 (12.9) years, body mass index 25.6 (3.6) kg/m2, and blood pressure 134.9 (20.7) mm Hg systolic and 80.4 (20.7) mm Hg diastolic. Mean values were 1.75 (1.15) mmol/L for triglycerides, 4.70 (1.08) and 1.08 (0.28) for total and HDL-cholesterol, respectively, and 97.3 (26.0) mL/min/1.73 m2 for eGFR. Geometric means were 1.03 (IQR, 0.69–1.58) for HOMA-IR and 0.40 nmol/L (IQR, 0.26–0.62) for C-peptide. Of the 798 diabetic patients, 605 (75.8%) had dyslipidaemia and 428 (53.6%) had hypertension. In all 1060 FLEMENGHO participants (50.6% women), age averaged 51.1 (15.6) years, body mass index 26.2 (4.2) kg/m2, and blood pressure 130.2 (17.0) mm Hg systolic and 80.8 (9.5) mm Hg diastolic. Mean values were 1.62 (0.88) mmol/L for triglycerides, 5.10 (0.93) and 1.48 (0.38) for total and HDL-cholesterol, respectively, and 86.1 (23.6) mL/min/1.73 m2 for eGFR. Geometric means were 0.64 (IQR, 0.40–0.95) for HOMA-IR and 34.5 pmol/L (IQR, 21.5–50.9) for insulin. Of the 1060 non-diabetic participants, 747 (70.5%) had dyslipidaemia and 331 (31.2%) had hypertension. S1 Table describes the use of drugs for diabetes, dyslipidaemia and hypertension by drug class in the two study populations.

### Unadjusted analyses

**Fig 1:**
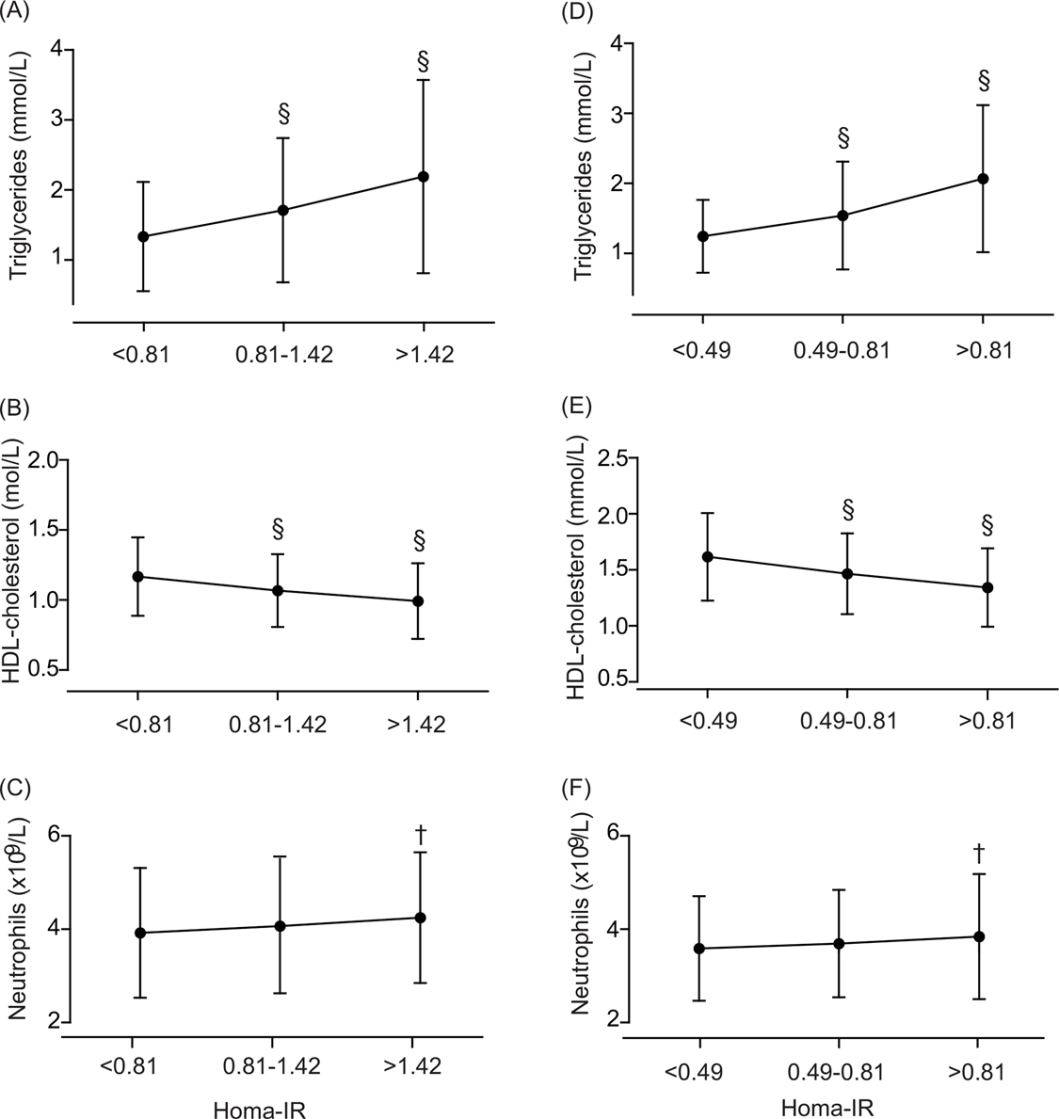
**Serum triglycerides (A, D), serum HDL-cholesterol (B, E) and neutrophil count (C, F) by thirds of the distribution of insulin resistance.** Insulin resistance was computed by Homeostasis Model Assessment algorithm (HOMA-IR), using C peptide in diabetic Lu Hepatients (A, B, C) patients and insulin in non-diabetic FLEMENGHO participants (D, E, F). Significance of the difference with the adjacent lower third: ɫ p≤0.01; § p≤0.0001.

We explored trends in the main variables across thirds of the distribution of the indexes of insulin resistance. In Lu He patients, the prevalence of dyslipidaemia (p = 0.0001), hypertension (p = 0.0027), history of cardiovascular disease (p = 0.0304) and renal dysfunction (p = 0.0002) increased with higher HOMA-IR category (Table 1). Body mass index (p<0.0001), diastolic blood pressure (p = 0.0053), serum triglycerides (p<0.0001) and both the white blood cell (p = 0.0014) and neutrophil counts (p = 0.030) increased with higher HOMA-IR, whereas HDL-cholesterol showed the opposite trend (p<0.0001). Among Flemish people, the prevalence of dyslipidaemia (p<0.0001), hypertension (p = 0.0011) and renal dysfunction (p = 0.0125) increased across thirds of the HOMA-IR distribution (Table 1). Body mass index, (p<0.0001), systolic blood pressure (p<0.0001), serum triglycerides (p<0.0001) and the neutrophil count (p = 0.019) increased with higher HOMA-IR, whereas HDL-cholesterol showed the opposite trend (p<0.0001). Analyses across thirds of the C-peptide distribution in Chinese diabetic patients and across thirds of the insulin distribution in non-diabetic Flemish produced results similar to those associated with HOMA-IR computed from these variables (S2 Table and Fig 1).

**Table 1:** Characteristics of diabetic patients and non-diabetic people by category of insulin resistance.

Characteristic	Type-2 diabetic Lu He patients			Non-diabetic FLEMENGHO participants		
	Category of insulin resistance			Category of insulin resistance		
Limits	<0.81	0.81–1.42	>1.42	<0.49	0.49–0.81	>0.81
N° in category (%)	265	267	266	352	355	353
Women	151 (57.0%)	147 (55.1%)	128 (48.1%)	194 (55.1%)	176 (49.6%)	166 (47.0%)
Hypertension	122 (46.0%)	144 (53.9%)	162 (60.9%)	90 (25.6%)	106 (30.0%)	135 (38.2%)*
Dyslipidaemia	179 (67.5%)	204 (76.4%)*	222 (83.5%)*	222 (63.1%)	243 (68.5%)	282 (79.9%)ǂ
Previous cardiovascular disease	45 (17.0%)	49 (18.4%)	68 (25.6%)*	25 (7.1%)	34 (9.6%)	34 (9.6%)
eGFR < 60 mL/min/1.73 m^2^	17 (6.4%)	18 (6.7%)	42 (15.8%)ǂ	37 (10.5%)	60 (16.9%)*	63 (17.9%)
Mean (SD)						
Age (years)	61.2 (12.9)	61.1 (11.9)	59.4 (13.)	49.6 (14.5)	51.54 (15.6)	52.3 (16.6)
Body mass index (kg/m^2^)	23.9 (3.3)	25.9 (3.3)§	26.9 (3.7)ɫ	23.9 (3.0)	26.0 (3.6)§	28.8 (4.2)§
Systolic pressure (mm Hg)	134.2 (22.7)	134.4 (17.9)	136.0 (21.3)	127.5 (16.6)	130.1 (17.6)*	133.0 (16.5)*
Diastolic pressure (mm Hg)	78.8 (11.6)	80.4 (10.7)	82.1 (10.9)*	79.5 (9.4)	80.5 (9.3)	82.5 (9.6)ɫ
Heart rate (beats per minute)	79.2 (10.3)	78.3 (12.1)	79.1 (11.0)	61.5 (8.6)	63.0 (8.9)*	65.6 (9.9)ǂ
Plasma glucose (mmol/L)	7.6 (2.7)	8.3 (3.0)ɫ	9.2 (3.4)ɫ	4.6 (0.4)	4.7 (0.4)ǂ	4.9 (0.6)ǂ
eGFR (mL/min/1.73 m^2^)	100.1 (22.3)	98.5(25.3)	93.1 (29.4)*	88.9 (21.1)	86.1 (24.7)	83.3 (24.6)*
Total cholesterol (mmol/L)	4.76 (1.06)	4.61 (1.04)	4.78 (1.14)	5.10 (0.90)	5.03 (0.91)	5.16 (0.98)
HDL cholesterol (mmol/L)	1.17 (0.28)	1.07 (0.26)§	0.99 (0.27)ǂ	1.62 (0.39)	1.46 (0.36)§	1.35 (0.35)§
LDL cholesterol (mmol/L)	2.98 (0.93)	2.84 (0.88)	2.96 (0.97)	2.92 (0.76)	2.87 (0.80)	2.87 (0.85)
Triglycerides (mmol/L)	1.33 (0.78)	1.71 (1.03)§	2.19 (1.38)§	1.25 (0.52)	1.55 (0.77)§	2.06 (1.05)§
White blood cell count (× 10^9^/L)	6.4 (1.6)	6.7 (1.7)*	6.9 (1.7)	6.3 (1.6)	6.3 (1.6)	6.5 (1.7)*
Neutrophils (%)	60.4 (10.1)	59.5 (10.1)	60.6 (9.4)	56.9 (7.7)	58.2 (7.9)*	58.1 (8.4)
Geometric mean (IQR)						
C-peptide (nmol/L)	0.19 (0.16–0.28)	0.42 (0.36–0.50)§	0.79 (0.61–0.96)§	…	…	…
Insulin (pmol/L)	…	…	…	18.3 (14.4–21.5)	33.7 (28.7–37.3)§	66.2 (50.9–78.2)§

Abbreviations: eGFR, estimated glomerular filtration rate derived from serum creatinine by Chronic Kidney Disease Epidemiology Collaboration (CKD-EPI) equation; HDL, high-density lipoprotein; LDL, low-density lipoprotein; IQR interquartile range. Insulin resistance was computed by Homeostasis Model Assessment algorithm (http://www. dtu.ox.ac.uk/HOMAcalculator/) using C-peptide in diabetic patients and insulin in non-diabetic people. Hypertension was a blood pressure of ≥140 mm Hg systolic or ≥90 mm Hg diastolic or use of antihypertensive drugs. Dyslipidaemia included total cholesterol >4.9 mmol/L, LDL-cholesterol >3 mmol/L, or triglycerides >1.7 mmol/L or HDL-cholesterol <1.2 mmol/L in women and <1 mmol/L in men. Significance of the difference with the adjacent lower third

* p≤0.05

ɫ p≤0.01

ǂ p≤0.001

§ p≤0.0001

An ellipsis indicates variable not measured.

### Multivariable-adjusted analyses

S3 Table list the covariables selected by stepwise regression to be accounted for in the multi-variable adjusted analyses. For continuous variables association sizes were standardised to a 1-SD increment in the explanatory variable. In Lu He patients, HOMA-IR increased with body mass index (effect size, +27.1%; p<0.0001) and mean arterial pressure (+5.9%; p = 0.018). HOMA-IR was also positively and independently associated with use of niacin (+16.8%; p = 0.039) and inversely with insulin treatment (-23.0%; p<0.0001). Use of β-blockers was weakly associated with HOMA-IR (+14.0%; p = 0.10). Together, these covariables explained 17.9% of the variance in the Chinese diabetic patients. In Flemish, HOMA-IR decreased with age (-6.6%; p<0.0001) but increased with body mass index (+35.8%; p<0.0001), use of statins (+16.1%; p = 0.0013), use of fibrates (+53.0%; p = 0.014) and use of β-blockers (+14.3%; p = 0.006). The independent and positive associations in Flemish of HOMA-IR with use of nonsteroidal anti-inflammatory drugs (+9.1%; p = 0.067) did not reach formal significance. Combined, these covariables explained 30.4% of the HOMA-IR variance in the non-diabetic Flemish.

In the next step of the analysis, we standardised HOMA-IR to the average (mean or ratio) of the aforementioned covariables identified by stepwise regression in the two study populations. Triglycerides, HDL-cholesterol and neutrophil count were first entered separately in the models (Table 2). In Chinese diabetic patients, HOMA-IR was positively associated with triglycerides and neutrophil count and inversely with HDL-cholesterol. Per 1-SD increment in the explanatory variable, the changes in HOMA-IR amounted to +11.7% (p<0.0001), +5.8% (p = 0.017) and -11.2% (p<0.0001). In Flemish non-diabetic participants, the corresponding estimates were +12.8% (p<0.0001), +3.8% (p = 0.0125) and -6.2% (p<0.0001), respectively. Models relating HOMA-IR to the three explanatory variables jointly produced similar results, although in Flemish participants the association between HOMA-IR and HDL-cholesterol weakened to -1.7% (p = 0.28). None of the interaction terms between the three explanatory variables reached significance in Chinese (p≥0.10) or Flemish (p≥0.10).

**Table 2:** Multivariable-adjusted associations of insulin resistance with serum lipids and neutrophil count.

Population Explanatory variable	HOMA-IR		Insulin index	
	Estimate (95% CI)	p	Estimate (95% CI)	p
Lu He patients				
Triglycerides (+1.15 mmol/L)	11.7 (6.8 to 16.9)	<0.0001	8.0 (3.4 to 12.9)	0.0006
HDL-cholesterol (+0.28 mmol/L)	-11.2 (-15.1 to -7.1)	<0.0001	-8.6 (-12.5 to -4.5)	<0.0001
Neutrophils (1.41 × 10^9^/L)	5.8 (1.0 to 10.7)	0.017	5.6 (1.1 to 10.4)	0.0151
FLEMENGHO participants				
Triglycerides (+0.88 mmol/L)	12.8 (9.4 to 15.8)	<0.0001	12.4 (9.3 to 15.7)	<0.0001
HDL-cholesterol (+0.38 mmol/L)	-6.0 (-8.7 to -3.3)	<0.0001	-5.8 (-8.5 to -3.1)	<0.0001
Neutrophils (1.21 × 10^9^/L)	3.8 (0.9 to 6.9)	0.0112	4.0 (1.1 to 7.1)	0.0074

Insulin index refers to C-peptide in Lu He patients and insulin in FLEMENGHO participants. Estimates express the percentage change in the dependent variable associated with a 1-SD increment in the explanatory variables. All estimates were standardised to the average in each population (mean or ratio) of age, body mass index, mean arterial pressure, and use of antidiabetic medications (by class), lipid-lowering drugs (by class), antihypertensive drugs (by class), and nonsteroidal anti-inflammatory drugs (see S3 Table). Each explanatory variable was entered separately in the models.

**Fig 2:**
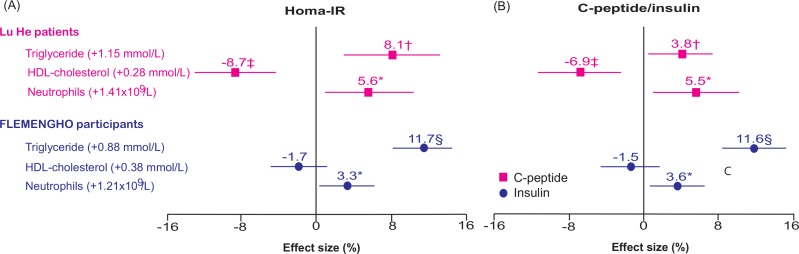
**Insulin resistance (A) and C-peptide or fasting insulin (B) in relation to serum lipids and neutrophil count in Lu He patients and FLEMENGHO participants.** Insulin resistance was computed by Homeostasis Model Assessment algorithm (HOMA-IR), using C peptide in diabetic Lu He patients (■) and fasting insulin (●) in non-diabetic FLEMENGHO participants. Estimates express the percentage change in the dependent variable associated with a 1-SD increment in the explanatory variables. All estimates were standardised to the average in each population (mean or ratio) of age, body mass index, mean arterial pressure, and use of antidiabetic medications (by class), lipid-lowering drugs (by class), antihypertensive drugs (by class), and nonsteroidal anti-inflammatory drugs (see S3 Table). The three explanatory variables were jointly entered in the models.

**Fig 3:**
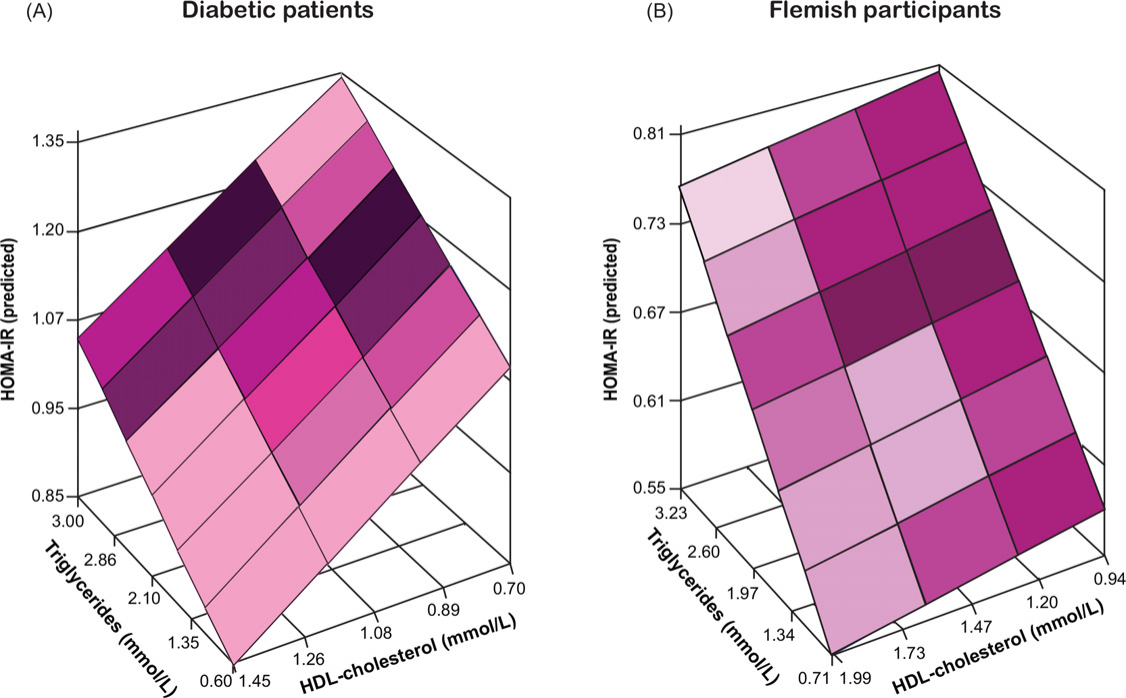
Insulin resistance as function of the serum levels of triglycerides and HDL-cholesterol and the neutrophil blood cell count. Insulin resistance was computed by Homeostasis Model Assessment algorithm (HOMA-IR) and plotted along the vertical axis. Serum triglycerides and HDL-cholesterol are plotted along the horizontal axes. The colour gradient in the modelled two-dimensional plane represent the gradient of the neutrophil blood cell count. All estimates were standardised to the average in each population (mean or ratio) of age, body mass index, mean arterial pressure, and use of antidiabetic medications (by class), lipid-lowering drugs (by class), antihypertensive drugs (by class), and nonsteroidal anti-inflammatory drugs (see S3 Table).

Analyses, in which HOMA-IR was substituted by C-peptide in Lu He patients or by insulin in Flemish, produced consistent results both in terms of selection of covariables for which the insulin indexes had to be standardised (S3 Table) as in terms of the associations with triglycerides, HDL-cholesterol or neutrophil count (Table 2 and Figs 2 and 3).

## Discussion

The key finding of the current study was that insulin resistance, as captured by the HOMA-IR index, increased with the serum level of triglycerides and the blood neutrophil count and decreased with serum HDL-cholesterol. These associations were independent of one another and consistent in Flemish and Chinese thereby spanning two ethnic groups and establishing a continuum ranging from a non-diabetic population sample to type-2 diabetic patients admitted to a tertiary referral hospital.

The idea to move to clinical studies in humans originated from our previous experiments in mice showing that hypercholesterolaemia induced by a high-fat diet enhanced proliferation of haematopoietic stem and progenitor cells (HSPC) and their subsequent differentiation to myeloid cells including neutrophils.[6] Furthermore, in HSPC isolated from mice and brought into culture, HDL-cholesterol inhibited differentiation to neutrophils but not to monocytes.[6] Other researchers demonstrated that higher HDL particle concentrations or HDL functionality promotes insulin secretion and pancreatic β cell survival, peripheral glucose uptake, and suppresses inflammation.[20] Moreover, there is a close link between lipoprotein metabolism, HDL-cholesterol and circulating levels of triglycerides, which is disturbed in diabetic patients or in the presence of insulin resistance.[20] Our experimental data explain why we investigated insulin resistance in relation to serum triglycerides and HDL-cholesterol and why we used neutrophil count as an index of inflammation. Several other investigators also proposed total or neutrophil white blood cell count as a marker of inflammation in diabetic patients, because these counts predict macro- and micro-vascular complications over and beyond classical risk factors.[21, 22]

With insulin resistance as outcome, our observational cross-sectional data cannot differentiate whether dyslipidaemia and inflammation are on a causal pathway to insulin resistance or develop as a consequence of insulin resistance. However, as reviewed elsewhere,[22] experimental evidence clearly demonstrates that under defined conditions inflammatory mediators alone can trigger insulin resistance in cells, experimental models and humans, suggesting that inflammation might be proximal to metabolic deterioration.[7] Furthermore, in the presence of excess of nutrients and energy substrate, metabolic signals likely activate inflammatory pathways, which then further disrupt metabolic function, leading to a vicious cycle involving further metabolic stress and inflammation.[22] Because we replicated our findings in non-diabetic people, we propose that in our current study dyslipidaemia and inflammation were chaperones proximal rather than secondary to insulin resistance. Our experimental studies[6] also support the hypothesis that high serum HDL-cholesterol is proximal to low insulin resistance.

The current study must be interpreted within the context of some potential limitations. First, as mentioned before, we cannot differentiate the association of insulin resistance with serum triglycerides and HDL-cholesterol or blood neutrophil count as cause or consequence. Second, we did not compare the enzymatic activity of circulating neutrophils in participants with low *vs*. high insulin resistance. Third, HDL functionality is exerted by the content of the lipoprotein particles, such as apolipoproteins,[23] enzymes,[23] or microRNAs.[24] Our epidemiological findings did not address how variation in the content of HDL-particles is related to insulin resistance. Finally, we only studied blood neutrophil count as established[9] marker of inflammation. To be addressed, to keep the head to head comparison, the classical CKD-EPI formula was applied to calculate eGFR in both cohorts. Alternatively, the classical modification of the CKD-EPI formula for Asian people could be more suitable for Chinese patients. Consistent with previous reports[25, 26], we observed in multivariable regression models higher values of HOMA-IR in Flemish participants taking statins or in Chinese patients taking niacin compared with those not receiving statin or niacin therapy.

The clinical implication of our study is that lowering serum triglycerides, increasing serum HDL-cholesterol and controlling inflammation might be important targets in the prevention or treatment of insulin resistance and its progression to type-2 diabetes mellitus. Intervention trials focused on controlling the inflammatory activity of monocytes in the treatment of diabetes.[27, 28] In type-2 diabetic patients, interleukin-1β receptor antagonists[27] and neutralising interleukin-1β antibodies[28] enhance C-peptide secretion[27] and reduce circulating levels of interleukin-6[27, 28] and C-reactive protein[27, 28], but have no effect on insulin resistance.[27] The null effect of interleukin-1β suppression on insulin resistance might be explained by the heterogeneity of macrophage subpopulations that exert opposite effects on insulin resistance[5] and the involvement of other cell types.[29, 30] Recently, experimental data described the role of neutrophils, T cells and monocytes in the peripheral organs of obese and diabetic mice.[30–32] Furthermore, in obese mice, deletion of myeloperoxidase or elastase in neutrophils prevented insulin receptor substrate 1 degradation and ameliorated pro-inflammatory cytokine production, resulting in the restoration of insulin signalling pathways, inhibition of monocyte infiltration of the liver and adipose tissues, and thus improved insulin sensitivity.[30–32] In line with these experimental mice studies, increased levels of elastase, myeloperoxidase and neutrophil count have also been observed in sera of obese human subjects.[31, 33] Therefore, controlling neutrophil count and recruitment of neutrophils could be an attractive target in the prevention and treatment of insulin resistance.

## Conclusions

In conclusion, our data demonstrated independent and positive associations of insulin resistance, as measured by HOMA-IR index with serum triglycerides and the blood neutrophil count, and an inverse association with serum HDL-cholesterol in both non-diabetic FLEMENGHO participants and Chinese type-2 diabetic patients. Our results were consistent across two ethnic groups and covered the continuum ranging from a non-diabetic population to type-2 diabetic patients. Building on our experimental studies,[6] our observations in humans suggest that lowering serum triglycerides, increasing serum HDL-cholesterol and controlling inflammation are potential targets for the prevention and treatment of insulin resistance, which might be universally and cumulatively applicable.

## Supporting Information

S1 Fig Flow chart. (EPS)

S1 Table The use of medications in type-2 diabetic patients and non-diabetic participants. (DOCX)

S2 Table Characteristics of diabetic patients and non-diabetic people by category of C-peptide or insulin. (DOC)

S3 Table Covariables selected by stepwise regression in type-2 diabetic patients and non-diabetic people. (DOC)
