# Supplementary material for: Insulin Resistance in Relation to Lipids and Inflammation in Type-2 Diabetic Patients and Non-Diabetic People
Source: PLoS One. 2016 Apr 13;11(4):e0153171. doi: 10.1371/journal.pone.0153171 (PMC4830613; doi:10.1371/journal.pone.0153171)
Supplement: S1 Table — (DOCX) [file pone.0153171.s002.docx]

**S1 Table.**

**The use of medications in type‑2 diabetic patients and non-diabetic participants**

| **Drugs** |  | **Lu He patients**  (n=798) |  | **FLEMENGHO  participants**  (n=1060) |  |
| --- | --- | --- | --- | --- | --- |
| Antidiabetic drugs |  |  |  |  |  |
| Insulin |  | 497 (62.3%) |  | ... |  |
| Metformin |  | 289 (36.2%) |  | ... |  |
| Sulfonylureas |  | 178 (22.3%) |  | ... |  |
| α-glucosidase inhibitors |  | 376 (47.1%) |  | ... |  |
| On ≥2 drug classes |  | 491 (61.5%) |  | ... |  |
| Lipid-lowering drugs |  |  |  |  |  |
| Niacin |  | 90 (11.3%) |  | 0 |  |
| Fibrates |  | 2 (0.3%) |  | 8 (0.8%) |  |
| Statins |  | 181 (22.7%) |  | 140 (13.2%) |  |
| On ≥2 drug classes |  | 1 (0.1%) |  | 4 (0.4%) |  |
| Antihypertensive drugs |  |  |  |  |  |
| Diuretics |  | 14 (1.8%) |  | 88 (8.3%) |  |
| β-blockers |  | 78 (9.8%) |  | 139 (13.1%) |  |
| Calcium-channel blockers |  | 139 (17.4%) |  | 58 (5.5%) |  |
| Angiotensin-converting enzyme inhibitors |  | 103 (12.9%) |  | 46 (4.3%) |  |
| Angiotensin receptor blockers |  | 86 (10.8%) |  | 27 (2.5%) |  |
| Other antihypertensive drugs |  | 0 |  | 4 (0.4%) |  |
| On ≥2 drug classes |  | 106 (13.3%) |  | 90 (8.5%) |  |
| NSAID |  | 548 (68.7%) |  | 136 (12.8%) |  |

NSAID indicates nonsteroidal anti-inflammatory drugs, including aspirin. An ellipsis indicates not applicable.
