# Supplementary material for: Insulin Resistance in Relation to Lipids and Inflammation in Type-2 Diabetic Patients and Non-Diabetic People
Source: PLoS One. 2016 Apr 13;11(4):e0153171. doi: 10.1371/journal.pone.0153171 (PMC4830613; doi:10.1371/journal.pone.0153171)
Supplement: S2 Table — (DOC) [file pone.0153171.s003.doc]

**S1 Table.**

**Characteristics of diabetic patients and non-diabetic people by category of C-peptide or insulin**

| **Characteristic** |  | **Type-2 diabetic Lu He patients** | | |  | **Non-diabetic FLEMENGHO participants** | | |
| --- | --- | --- | --- | --- | --- | --- | --- | --- |
|  |  | Category of C-peptide (nmol/L) | | |  | Category of insulin (pmol/L) | | |
| Limits |  | <0.32 | 0.32–0.55 | >0.55 |  | <27.28 | 27.28–43.23 | ≥43.23 |
| N° in category (%) |  | 265 | 267 | 266 |  | 352 | 355 | 353 |
| Women |  | 148 (55.9%) | 147 (55.1%) | 131 (49.1%) |  | 195 (55.4%) | 175 (49.3%) | 166 (47.0%) |
| Hypertension |  | 113 (42.6%) | 150 (56.2%) ɫ | 165 (62.0%) |  | 89 (25.3%) | 106 (29.9%) | 136 (38.5%)* |
| Dyslipidaemia |  | 183 (69.1%) | 201 (75.3%) | 221 (83.1%)* |  | 222 (63.1%) | 246 (69.3%) ɫ | 279 (79.0%) ɫ |
| Previous cardiovascular disease |  | 48 (18.1%) | 42 (15.7%) | 72 (27.1%) ɫ |  | 25 (7.1%) | 33 (9.3%) | 35 (9.9%) |
| eGFR < 60 mL/min/1.73 m^2^ |  | 15 (5.7%) | 20 (7.5%) | 42 (15.8%) ɫ |  | 37 (10.5%) | 61 (17.2%)* | 62 (17.6%) |
| Mean (SD) |  |  |  |  |  |  |  |  |
| Age (years) |  | 60.8 (12.8) | 61.3 (12.0) | 59.7 (13.7) |  | 49.6 (14.5) | 51.8 (15.4) | 52.0 (16.8) |
| Body mass index (kg/m^2^) |  | 23.9 (3.3) | 25.7 (3.2)§ | 27.0 (3.6)§ |  | 23.9 (3.0) | 25.9 (3.5)§ | 28.8 (4.3)§ |
| Systolic pressure (mm Hg) |  | 133.3 (22.3) | 135.3 (18.5) | 136.0 (21.2) |  | 127.4 (16.4) | 129.9 (17.5) | 133.1 (16.6) ɫ |
| Diastolic pressure (mm Hg ) |  | 78.6 (11.5) | 81.0 (9.8) * | 81.7 (11.8) |  | 79.5 (9.4) | 80.6 (9.4) | 82.5 (9.6) * |
| Heart rate (beats per minute) |  | 79.8 (10.4) | 78.2 (11.7) * | 78.5 (11.2) |  | 61.4 (8.5) | 63.0 (9.0) * | 65.6 (9.9) ǂ |
| Plasma glucose (mmol/L) |  | 8.4 (3.2) | 8.5 (3.2) | 8.1 (2.9) |  | 4.6 (0.4) | 4.8 (0.5) ǂ | 4.9 (0.6) ɫ |
| eGFR (mL/min/1.73 m^2^) |  | 101.0 (22.6) | 97.9 (24.3) ǂ | 92.9 (29.9) * |  | 89.0 (21.1) | 85.5 (24.4) | 83.8 (24.9) |
| total cholesterol (mmol/L) |  | 4.73 (1.04) | 4.67 (1.07) | 4.74 (1.14) |  | 5.10 (0.89) | 5.05 (0.92) | 5.13 (0.98) |
| HDL cholesterol (mmol/L) |  | 1.14 (0.28) | 1.09 (0.28) * | 1.00 (0.27)§ |  | 1.62 (0.39) | 1.45 (0.35)§ | 1.36 (0.36) ǂ |
| LDL cholesterol (mmol/L) |  | 2.95 (0.90) | 2.89 (0.90) | 2.94 (0.97) |  | 2.92 (0.76) | 2.88 (0.81) | 2.85 (0.85) |
| Triglyceride (mmol/L) |  | 1.43 (0.92) | 1.72 (1.06)§ | 2.09 (1.32)§ |  | 1.24 (0.52) | 1.59 (0.81)§ | 2.03 (1.03)§ |
| White blood cell count (× 10^9/L^) |  | 6.4 (1.6) | 6.7 (1.7) * | 6.9 (1.7) |  | 6.3 (1.6) | 6.3 (1.6) | 6.5 (1.7) |
| Neutrophils (%) |  | 60.2 (10.1) | 60.1 (10.2) | 60.3 (9.3) |  | 56.9 (7.7) | 58.2 (7.8) * | 58.3 (8.4) |

Abbreviations: eGFR, estimated glomerular filtration rate derived from serum creatinine by Chronic Kidney Disease Epidemiology Collaboration (CKD-EPI) equation; HDL, high-density lipoprotein; LDL, low-density lipoprotein; IQR interquartile range. Insulin resistance was computed by Homeostasis Model Assessment algorithm ([http: //www. dtu.ox.ac.uk/HOMAcalculator/](http://www.dtu.ox.ac.uk/homacalculator/)) using C‑peptide in diabetic patients and insulin in non-diabetic people. Hypertension was a blood pressure of ≥140 mm Hg systolic or ≥90 mm Hg diastolic or use of antihypertensive drugs. Dyslipidaemia included total cholesterol >4.9 mmol/L, LDL-cholesterol >3 mmol/L, or triglycerides >1.7 mmol/L or HDL-cholesterol <1.2 mmol/L in women and <1 mmol/L in men. Significance of the difference with the adjacent lower third: * p≤0.05; ɫ p≤0.01; ǂ p≤0.001; and § p≤0.0001. An ellipsis indicates variable not measured.
