# Supplementary material for: Insulin Resistance in Relation to Lipids and Inflammation in Type-2 Diabetic Patients and Non-Diabetic People
Source: PLoS One. 2016 Apr 13;11(4):e0153171. doi: 10.1371/journal.pone.0153171 (PMC4830613; doi:10.1371/journal.pone.0153171)
Supplement: S3 Table — (DOC) [file pone.0153171.s004.doc]

**S1 Table.**

**Covariables selected by stepwise regression in type‑2 diabetic patients and non-diabetic people**

| **Variables** |  | **Lu He patients** | | |  | **FLEMENGHO participants** | | |
| --- | --- | --- | --- | --- | --- | --- | --- | --- |
|  |  | HOMA‑IR |  | C‑peptide |  | HOMA‑IR |  | Insulin |
| R2 |  | 0.179 |  | 0.182 |  | 0.304 |  | 0.308 |
| Age (years) |  | ... |  | ... |  | ‑6.6 (‑9.8 to ‑3.3) ǂ |  | ‑7.4 (‑10.6 to ‑4.0) ǂ |
| Body mass index (kg/m2) |  | 27.1 (21.2 to 33.2) ǂ |  | 26.9 (21.2 to 32.8) ǂ |  | 35.8 (31.7 to 40.0) ǂ |  | 35.7 (30.6 to 39.9) ǂ |
| Mean arterial pressure (mm Hg) |  | 5.9 (1.2 to 10.7)* |  | 5.5 (0.9 to 10.3) * |  | ... |  | ... |
| Use of medications |  |  |  |  |  |  |  |  |
| Insulin (0, 1) |  | ‑23.0 (‑30.1 to ‑15.2) ǂ |  | ‑24.3 (‑31.0 to ‑16.8) ǂ |  | NA |  | NA |
| Statins (0, 1) |  | ... |  | ... |  | 16.1 (6.0 to 27.2) ɫ |  | 16.4 (6.2 to 27.5) ɫ |
| Niacin (0, 1) |  | 16.8 (0.8 to 35.4)* |  | ... |  | ... |  | ... |
| Fibrates (0, 1) |  | ... |  | ... |  | 53.0 (8.9 to 115.0) * |  | 52.2 (8.3 to 114.1) * |
| β‑blockers (0, 1) |  | 14.0 (‑2.6 to 33.4) |  | 14.5 (‑1.6 to 33.3) |  | 14.3 (3.9 to 25.7) ɫ |  | 13.8 (3.4 to 25.2) ɫ |
| Diuretics (0, 1) |  | ... |  | ... |  | 8.8 (‑2.9 to 21.9) |  | 8.8 (‑2.9 to 21.9) |
| Aspirin or NSAID (0,1) |  | ... |  | ... |  | 9.1 (‑0.6 to 19.7) |  | 9.3 (‑0.4 to 19.9) |

p‑values for entering and retaining covariables in the models were set at 0.15. Covariables considered included sex, age, body mass index, mean arterial pressure, use of antidiabetic drugs (insulin, sulfonylurea, metformin and α‑ glucosidase inhibitors), lipid-lowering drugs (statins, niacin and fibrates), antihypertensive drugs (diuretics, β‑blockers, calcium channel blockers and inhibitors of the renin-angiotensin system [angiotensin-converting enzyme inhibitors and angiotensin receptor blockers]) and aspirin. HOMA‑IR, C‑peptide and insulin were logarithmically transferred. Insulin resistance was computed by Homeostasis Model Assessment (http://www.dtu.ox.ac.uk/homacalculator/), using C‑peptide in diabetic patients and insulin in non-diabetic people. For continuous variables, the association sizes are expressed for 1‑SD increase. Association sizes (95% confidence interval) express the percentage change in the indexes of insulin resistance per 1‑SD increase in the continuous variables or for a condition being present *vs.* absent. Significance of the associations: * p≤0.05; ɫ p≤0.01; ǂ p≤0.001; and § p≤0.0001. An ellipsis indicates that a covariable did not enter the model. NA means not applicable.
